# Shared Pathophysiology and Early Detection Biomarkers in Endometriosis and Polycystic Ovary Syndrome (PCOS): Opportunities for AI-Enabled Screening

**DOI:** 10.7759/cureus.106013

**Published:** 2026-03-28

**Authors:** Shubham Gupta, Anushree, Goter Doke, Devyani Misra, Ashish Kumar Shukla, B. Honey

**Affiliations:** 1 Department of Radio-diagnosis, Jammu University, Jammu, IND; 2 Department of Obstetrics and Gynecology, Government Medical College, Haldwani, Haldwani, IND; 3 Department of Obstetrics and Gynecology, Tomo Riba Institute of Health and Medical Sciences, Naharlagun, IND; 4 Department of Obstetrics and Gynecology, Dr. Ram Manohar Lohia Institute of Medical Sciences, Lucknow, IND; 5 Department of Radio-diagnosis, Santosh Medical College, Santosh Deemed to be University, Ghaziabad, IND; 6 Department of Pharmacology, Sree Balaji Medical College and Hospital, Chennai, IND

**Keywords:** artificial intelligence, early detection, endometriosis, polycystic ovarian syndrome, precision screening

## Abstract

Endometriosis and polycystic ovary syndrome (PCOS) are common, multifactorial gynecological disorders shaped by endocrine imbalance, immune dysfunction, metabolic disruption, genetic susceptibility, and environmental exposures. Despite their major contribution to infertility and long-term cardiometabolic morbidity, early detection remains poor because symptoms are nonspecific, phenotypes are heterogeneous, and diagnosis is still dominated by single-modality and symptom-driven pathways. This review addresses this gap by synthesizing 2015-2025 evidence on shared and disease-specific biological mechanisms and evaluating how artificial intelligence (AI) can improve scalable screening and risk stratification. A narrative and integrative methodology was applied using peer-reviewed studies retrieved from PubMed, Scopus, Web of Science, and Google Scholar, emphasizing diagnostic rigor and external validity. Key findings identify convergent pathways involving chronic low-grade inflammation, adipokine dysregulation, oxidative stress, microbiome-mediated estrogen signaling, ferroptosis-linked iron imbalance, mitochondrial dysfunction, and epigenetic regulation through microRNAs (miRNAs) and long non-coding RNAs (lncRNAs). Promising early-detection signals include age-stratified anti-Müllerian hormone (AMH) thresholds, circulating cell-free deoxyribonucleic acid (cfDNA) methylation markers, and reproductive tract microbial signatures. AI-based models, including transformer architectures and multimodal machine learning, show strong potential to integrate clinical, hormonal, imaging, omics, and digital symptom phenotyping into reproducible early screening frameworks. Clinical translation requires standardized diagnostic definitions, longitudinal multi-ethnic cohorts, explainable algorithms, and prospective validation. AI-enabled precision screening offers a practical pathway to shorten diagnostic delay and improve reproductive outcomes.

## Introduction and background

Endometriosis and polycystic ovary syndrome (PCOS) are complex and multifactorial gynecological disorders that arise from interactions among endocrine dysregulation, immune dysfunction, metabolic imbalance, and environmental factors [1]. Low-grade chronic inflammation is a persistent, mild, systemic inflammatory condition that disrupts immune homeostasis and contributes to the development of several chronic diseases [1]. This inflammatory state is increasingly recognized as a contributing factor in reproductive disorders because of its effects on hormonal signaling, tissue remodeling, and immune surveillance [2]. Persistent inflammatory stimulation creates a pathological microenvironment that promotes disease persistence and chronic symptoms in gynecological conditions [1,3].

Reproductive disorders in women continue to represent a substantial global health burden [2,4]. Ovarian cancer is the second most common cancer affecting women and is the fifth leading cause of cancer-related deaths among women [5]. This epidemiological pattern highlights the susceptibility of ovarian and reproductive tissues to long-term pathological changes associated with inflammation, metabolic stress, and endocrine dysregulation [1,2]. The biological processes involved in benign gynecological disorders share similarities with those implicated in malignant transformation, underscoring the importance of early detection of pathological changes [6,7].

Despite the high global burden of reproductive disorders, early diagnostic tools for conditions such as endometriosis and PCOS remain limited. Many patients experience substantial delays in diagnosis because current clinical approaches rely heavily on symptom-based assessment and single-modality diagnostic methods. These limitations highlight the need for integrative screening strategies that combine biological markers, clinical indicators, and emerging computational approaches. In this context, artificial intelligence (AI) has emerged as a promising tool for analyzing complex biomedical data and supporting earlier detection and risk stratification in reproductive diseases.

Lifestyle and environmental factors also influence reproductive health outcomes [8,9]. Nutrition and dietary patterns can affect inflammatory signaling, endocrine balance, and metabolic regulation, which are relevant to the pathophysiology of endometriosis and PCOS [1,3]. Unfavorable dietary patterns may contribute to chronic inflammation and metabolic disturbances that exacerbate disease progression [1,3].

Infertility is a major global health concern with significant reproductive and psychosocial consequences [4,6]. According to the World Health Organization (WHO), approximately one in six individuals of reproductive age experiences infertility during their lifetime [4]. Endometriosis and PCOS are major contributors to female infertility because they are associated with ovulatory dysfunction, hormonal abnormalities, and inflammatory damage to reproductive tissues [4,6]. Diagnostic delays and the limited availability of early screening approaches further worsen infertility outcomes associated with these disorders [2,4].

Micronutrient balance is also important for maintaining systemic and reproductive health. Iron is an essential trace element required for oxygen transport, deoxyribonucleic acid (DNA) synthesis, cellular respiration, and immune function [5]. Disturbances in iron homeostasis may contribute to oxidative stress and inflammatory processes that affect reproductive tissue function [1,5].

**Figure 1:**
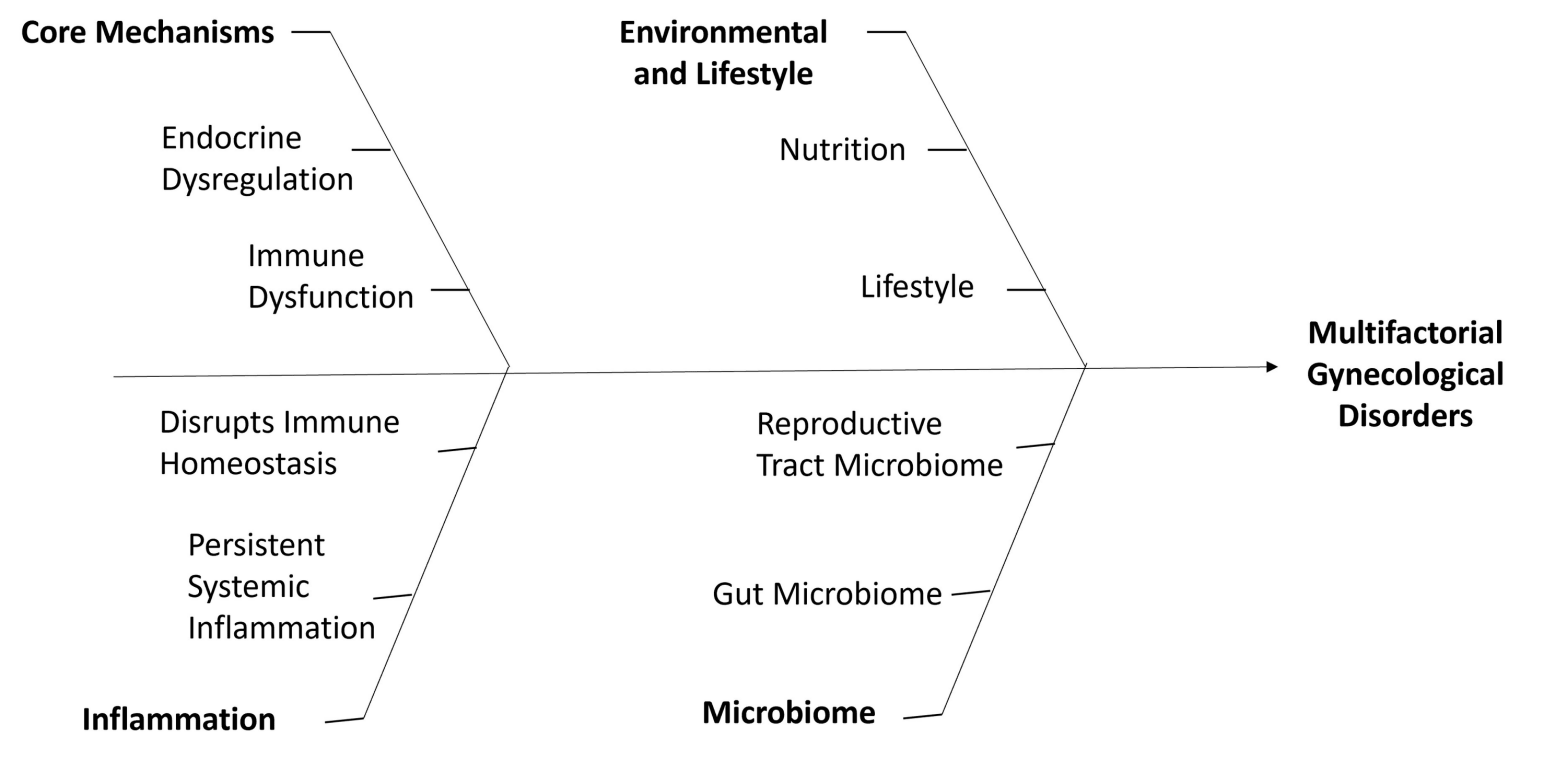
Multifactorial drivers of gynecological disorders Schematic representation of the multifactorial pathophysiology underlying gynecological disorders, illustrating the interaction between core biological mechanisms, environmental and lifestyle factors, and microbiome dynamics. The diagram depicts how endocrine dysregulation and immune dysfunction contribute to disrupted immune homeostasis and persistent systemic inflammation, while external influences such as nutrition and lifestyle modulate both host physiology and microbial composition. Alterations in the gut and reproductive tract microbiome further interact with inflammatory and immune pathways, collectively driving the development and progression of multifactorial gynecological disorders. The figure was created by the authors of this study using Microsoft PowerPoint (Microsoft Corporation, Redmond, Washington, United States).

The endometrium is one of the most dynamic adult tissues in the body, undergoing continuous cycles of growth, differentiation, shedding, and regeneration under strict hormonal regulation [6]. This dynamic remodeling makes the endometrium highly sensitive to inflammatory stimuli, metabolic stress, and hormonal imbalance [1,6]. Abnormal endometrial responses play a key role in the pathogenesis of endometriosis and are associated with implantation failure and persistent pelvic inflammation [4,6]. Figure 1 illustrates the complex etiology of gynecological disorders, which arises from interactions among endocrine, immune, microbiome, and lifestyle factors.

The female reproductive tract also hosts a specialized microbial ecosystem that contributes to mucosal immunity, epithelial barrier maintenance, and regulation of local inflammatory responses [7,9]. Alterations in microbial composition have been associated with adverse reproductive outcomes and inflammatory gynecological conditions [6,7].

Systemic microbial activity also influences reproductive endocrinology and hormonal homeostasis [8,9]. The gut microbiome, particularly the estrobolome, participates in estrogen metabolism by regulating estrogen circulation and bioavailability [8]. Dysregulation of these microbial processes may alter hormone exposure in estrogen-sensitive tissues such as the ovaries and endometrium, potentially contributing to endocrine imbalance and inflammation [1,8]. Microbial dysbiosis has also been linked to systemic inflammation and metabolic dysfunction associated with endometriosis and PCOS [3,9].

Metabolic signaling pathways also contribute to reproductive endocrine regulation. Inositols are six-membered cyclic polyols that function as mediators of intracellular signaling, particularly in insulin-related pathways [10]. Disturbances in inositol metabolism have been associated with insulin resistance and ovarian follicular dysfunction, both characteristic features of PCOS [4,10]. Taken together, inflammatory, metabolic, microbial, and hormonal mechanisms interact to influence the pathophysiology of reproductive disorders. Understanding these interconnected pathways may support the development of improved screening strategies that incorporate biomarker discovery and AI-assisted analytical approaches.

Objectives of the review

This review synthesizes evidence published between 2015 and 2025 regarding the shared inflammatory, endocrine, metabolic, genetic, and epigenetic pathways involved in endometriosis and polycystic ovarian syndrome. It critically evaluates the limitations of symptom-based and single-modality diagnostic approaches and identifies key biological markers that may enable earlier disease detection. The review also examines the potential role of AI in integrating multimodal datasets to improve screening, risk stratification, and diagnostic consistency. Finally, it discusses the translational potential of AI-based frameworks for developing personalized and scalable early detection strategies in reproductive health.

## Review

Methodology

Literature Search Strategy

This narrative review synthesized current evidence on shared biological mechanisms, biomarkers, and AI-based approaches for the early detection of endometriosis and PCOS. A structured search of electronic databases, including PubMed, Scopus, Web of Science, and Google Scholar, was conducted to identify relevant studies published between 2015 and 2025. The search strategy used combinations of keywords such as “endometriosis,” “polycystic ovary syndrome,” “PCOS,” “biomarkers,” “epigenetics,” “microRNA,” “artificial intelligence,” “machine learning,” “early detection,” and “reproductive disorders.” Reference lists of relevant articles were also screened to identify additional studies related to the topic. Identified studies were screened based on titles and abstracts, followed by full-text evaluation to determine relevance to the objectives of the review.

Inclusion and Exclusion Criteria

Studies were included if they investigated molecular mechanisms, genetic or epigenetic pathways, biomarkers, epidemiological factors, or AI applications related to the detection, pathophysiology, or risk stratification of endometriosis and PCOS. Both clinical studies and experimental investigations (in vitro and in vivo) were considered, along with relevant review articles that provided mechanistic or methodological insights. Articles not written in English, conference abstracts without full text, studies lacking sufficient methodological description, and publications not directly related to reproductive disorders, biomarker discovery, or diagnostic modeling were excluded.

Evidence Appraisal and Data Synthesis

Evidence appraisal focused on evaluating study design, methodological robustness, biological mechanisms investigated, and relevance to biomarker discovery or diagnostic modeling in reproductive disorders. Data from eligible studies were synthesized qualitatively to summarize shared inflammatory, endocrine, metabolic, genetic, and epigenetic mechanisms associated with endometriosis and PCOS, as well as emerging AI-based analytical approaches for disease screening, risk stratification, and early detection. Because the included studies differed substantially in design, study populations, and reported outcomes, quantitative meta-analysis was not performed, and findings were synthesized narratively.

Micronutrients

The combination of micronutrients and bioactive compounds contributes to female reproductive physiology through their roles in inflammatory regulation, metabolic balance, and ovarian function, with several micronutrients showing clinically relevant associations with reproductive biomarkers and endocrine regulation [11]. Zinc supplementation at doses of 20-30 mg/day has been shown to reduce the intensity and duration of menstrual pain in women with primary dysmenorrhea, providing clinical evidence for its association with inflammatory mediators that are investigated as potential biomarkers of reproductive pathology [11]. In addition to symptomatic effects, micronutrients influence ovarian cellular pathways related to follicular viability and oxidative balance, which are increasingly explored in biomarker research [12]. Ascorbic acid (vitamin C) promotes extracellular matrix reorganization and supports the survival of primary ovarian follicles in three-dimensional culture systems, suggesting involvement in pathways associated with ovarian tissue remodeling markers [12].

Additional micronutrients with clinical relevance include vitamin D and inositols, which are frequently studied in women with PCOS [13]. Marginal vitamin D levels have been associated with increased insulin resistance and elevated anti-Müllerian hormone (AMH) levels in women with PCOS, indicating dysfunctional follicular dynamics based on observational clinical evidence and highlighting AMH as an important hormonal biomarker in PCOS pathophysiology [14]. Vitamin D receptor genetic polymorphisms, particularly the TaqI variant, have also been linked to increased susceptibility to female infertility in population studies and may therefore represent potential genetic biomarkers for reproductive endocrine disorders [15]. Other metabolic regulators, such as D-chiro-inositol, enhance insulin signaling and reduce aromatase expression, thereby helping to regulate androgen-to-estrogen balance and influencing endocrine biomarkers involved in metabolic and reproductive regulation [16].

Disruption of micronutrient balance can lead to pathological alterations in the ovaries, with both clinical and mechanistic studies suggesting an important role for micronutrient homeostasis in ovarian health and in the regulation of oxidative stress markers associated with reproductive disease progression [17]. Pathological iron overload has been associated with ferroptosis and fibrosis of ovarian tissue, which may contribute to ovarian dysfunction in endometriosis and reproductive aging and may generate iron-related molecular signatures that serve as emerging biomarkers of ovarian pathology [18]. Overall, micronutrients such as vitamin D, zinc, and inositols demonstrate clinically relevant associations with reproductive and metabolic regulation, whereas several mechanistic insights regarding the ovarian follicular microenvironment and oxidative stress regulation remain primarily supported by experimental and preclinical studies that provide context for identifying hormonal, metabolic, and oxidative biomarkers in reproductive disorders [3,5,10-12].

Genetic, molecular, and DNA repair mechanisms in endometriosis and PCOS

DNA Repair Pathways and Genetic Susceptibility

Genetic, molecular, and metabolic dysfunctions represent important pathogenic mechanisms in endometriosis and PCOS and provide a foundation for biomarker identification and disease stratification [19]. RAD51 regulators, including XRCC3, BRCA1, and CSB, have been reported to exhibit genetic variation that may function as molecular markers associated with endometriosis susceptibility [19]. BRCA1 has also been implicated in the pathogenesis of PCOS, suggesting potential overlap in DNA repair pathway alterations between these reproductive disorders [19]. Genetic susceptibility may further interact with endocrine regulation mediated by micronutrients [20]. Vitamin D acts as an important regulator of ovarian steroidogenesis and follicular maturation through modulation of AMH and follicle-stimulating hormone receptor expression, both recognized as endocrine biomarkers of ovarian function [20].

Autoimmune-Endocrine Interactions and Mitochondrial Dysfunction

Autoimmune-endocrine interactions may also contribute to disease-specific molecular mechanisms [21]. Endometriosis has been associated with hyperthyroidism mediated by thyrotropin receptor antibodies, whereas PCOS has been linked to autoimmune hypothyroidism characterized by thyroid peroxidase and thyroglobulin antibodies, which serve as autoimmune biomarkers in endocrine disorders [21]. At the cellular level, mitochondrial dysfunction plays a significant role in metabolic dysregulation [22]. Impaired oxidative phosphorylation and mutations in mitochondrial DNA increase the generation of reactive oxygen species, representing key molecular indicators of oxidative stress observed in PCOS [22].

Oxidative Stress, Iron Metabolism, and Developmental Biomarkers

Conceptually, PCOS and endometriosis have been proposed as possible diametric disorders of the hypothalamic-pituitary-gonadal axis, characterized by contrasting patterns of prenatal testosterone exposure, anogenital distance, and AMH concentrations that may function as developmental and hormonal biomarkers distinguishing these conditions [18]. Iron overload in ovarian and gestational tissues may trigger ferroptosis, contributing to follicular atresia and placental dysfunction and representing a potential iron-related molecular pathway associated with reproductive disease biomarkers [5]. Vitamin D receptor polymorphisms, particularly the TaqI variant, have been identified as genetic risk factors associated with increased susceptibility to female infertility [4].

Mechanistic studies also indicate that inflammatory mediators such as matrix metalloproteinase-9 contribute to the progression of endometriosis, highlighting potential molecular targets relevant to disease monitoring [13]. Converging oxidative stress and iron-mediated toxicity contribute to reproductive tissue pathology, while environmental toxins and chronic inflammation may further exacerbate mitochondrial dysfunction and tissue scarring through pathways reflected in metabolic and inflammatory biomarker profiles [1,5,17,22]. Interactions among vitamin D status, microbial metabolism, and thyroid function may influence hormonal and metabolic homeostasis in the female reproductive tract and may ultimately affect oocyte quality through endocrine and metabolic biomarker pathways [7,8,20,21]. Table 1 summarizes shared genetic features and distinct clinical considerations in endometriosis and PCOS.

**Table 1:** Genetic, molecular features, and therapeutic approaches This table was created by the authors based on a synthesis of data from the cited references [2,5,6,13,15,19,23]. PCOS: polycystic ovary syndrome; GnRH: gonadotropin-releasing hormone; LNG-IUS: levonorgestrel-releasing intrauterine system; miRNA: microRNA; DNA: deoxyribonucleic acid

Disorder	Parameter	Description	References
Endometriosis	Genetic & Molecular Markers	Moderate heritability with variants in XRCC3, BRCA1, CDKN2B-AS1, WNT4, GREB1, ESR1, and CYP19A1; upregulation of ERα/ERβ and downregulation of PR-A/PR-B	[19,23]
	Therapeutic Approaches	GnRH agonists, progestins, LNG-IUS, surgical intervention, antioxidants; adjunctive use of curcumin and vitamin D to reduce lesion burden and inflammation	[2,5,13,15]
PCOS	Genetic & Molecular Markers	High heritability with variants in DENND1A, THADA, LHCGR, FSHR, VDR, and BRCA1; dysregulated miRNAs and mitochondrial dysfunction	[19,23]
	Therapeutic Approaches	Lifestyle modification, insulin sensitisers, combined oral contraceptives, anti-androgens, ovulation induction agents, and metabolic adjuncts such as curcumin	[2,6,13]

Shared genetic architecture and endocrine dysregulation in PCOS and endometriosis

Genetic and endocrine overlap between endometriosis and PCOS has been reported and may contribute to shared pathophysiological features between the two conditions [23]. Mendelian randomization analyses indicate a positive genetic correlation (rg = 0.56), suggesting the presence of shared heritable risk factors and potential bidirectional biological relationships [23]. This overlap is further reflected in endocrine disturbances, where stepwise multiple regression models have identified PCOS as a primary predictor of altered systemic concentrations of leptin, adiponectin, resistin, and ghrelin, independent of body mass index [24]. Elevated body mass index is also recognized as a major global risk factor contributing to mortality and disability-adjusted life years associated with several gynecological diseases [25]. Adipokines are multifunctional signaling molecules involved in energy metabolism and adipose tissue regulation and may contribute mechanistically to the pathogenesis of PCOS and endometriosis [26].

In some evolutionary and developmental frameworks, PCOS and endometriosis have been proposed as diametric disorders of the hypothalamic-pituitary-gonadal axis, characterized by opposing patterns of prenatal testosterone exposure and adult AMH levels; however, this hypothesis remains debated, and alternative explanations emphasize multifactorial genetic, metabolic, and environmental mechanisms underlying the pathogenesis of both conditions [18]. At the molecular level, alterations in the BRCA1 gene have been associated with reduced AMH levels and impaired DNA repair mechanisms, suggesting a potential role in both disorders [19]. Distinct autoimmune associations have also been reported, with endometriosis linked to Graves-related thyroid antibodies and PCOS more frequently associated with markers related to Hashimoto’s thyroiditis [21]. Mitochondrial DNA mutations and impaired oxidative phosphorylation may contribute to metabolic and hormonal alterations observed in PCOS [22]. Integration of genetic and developmental evidence has suggested a shared architecture involving pleiotropic loci and DNA repair regulators that may influence systemic hormonal sensitivity [18,19,27]. In addition, chronic low-grade inflammation, dysregulated adipokine signaling, and iron-mediated ferroptosis have been implicated as pathological mechanisms affecting the female reproductive tract [1,5,24,26].

Hormonal receptors, adipokines, and inflammatory mediators in disease pathogenesis

One of the characteristic features of the pathogenesis of endometriosis is an imbalance in hormonal receptor expression [27]. Ovarian function is also regulated by chemokine-mediated signaling pathways that influence steroidogenesis [28]. Immune dysregulation within the endometrium has been identified as an important feature associated with PCOS [29]. Developmental endocrine factors may further contribute to population-level susceptibility to these disorders [30]. Inflammatory signaling pathways interact with metabolic and endocrine mediators during disease progression [26]. Clinically, the diagnosis of PCOS has been identified as a strong predictor of alterations in systemic metabolic hormone profiles [24]. Pro-inflammatory cytokines may contribute to ovarian dysfunction by promoting fibrosis and oxidative stress [1]. Vitamin D signaling also plays an important role in regulating endometrial activity and embryo implantation [20]. Overall, hyperinsulinemia and hyperandrogenism may amplify inflammatory responses, suggesting that chronic reproductive disorders represent manifestations of interacting hormonal and immune dysregulation [21]. Table 2 summarizes the differing endocrine, metabolic, and immune regulatory patterns observed in endometriosis and PCOS.

**Table 2:** Endocrine, metabolic, and immune signatures in endometriosis and PCOS This table was created by the authors based on a synthesis of data from the cited references [1,20,21,24,26-29,30]. PCOS: polycystic ovary syndrome; BMI: body mass index; TNF-α: tumour necrosis factor alpha; IL-6: interleukin-6; HOXA10: homeobox A10; CXCL14: C-X-C motif chemokine ligand 14; FGF21: fibroblast growth factor 21; CD56+: cluster of differentiation 56 positive; P38: p38 mitogen-activated protein kinase; JNK: c-Jun N-terminal kinase

Disorder	Parameter	Description	References
Endometriosis	Sex steroid receptor balance	Hyperestrogenic molecular environment driven by upregulation of estrogen receptors with concurrent downregulation of progesterone receptors, resulting in impaired endometrial responsiveness.	[27]
	Chemokine-mediated steroidogenesis	In human luteinized granulosa cells, CXCL14 mediates progesterone synthesis by activating P38 and JNK pathways and increasing expression of steroidogenic acute regulatory protein.	[28]
PCOS	Endometrial immune profile	Secretory endometrium shows a significantly reduced proportion of CD56+ natural killer cells in infertile women compared with fertile controls, indicating compromised immune recruitment and diminished endometrial receptivity.	[29]
	Developmental endocrine influence	Populations of African ancestry show higher serum testosterone concentrations during pregnancy alongside a higher prevalence of PCOS, supporting a developmental link to elevated prenatal androgen exposure.	[30]
	Metabolic endocrine mediator	Batokine FGF21 acts as a cold-induced endocrine activator that improves adipose tissue insulin sensitivity and may ameliorate polycystic ovarian phenotypes in animal models.	[26]
	Systemic adipokines (clinical)	PCOS diagnosis is the primary predictor of altered systemic leptin, adiponectin, and ghrelin levels, independent of BMI.	[24]
Endometriosis + PCOS	Inflammatory mediators	TNF-α and IL-6 contribute to ovarian dysfunction by inducing tissue fibrosis and increasing oxidative stress in the follicular microenvironment.	[1]
	Endometrial implantation marker	Adequate vitamin D levels correlate with increased HOXA10 expression required for successful embryo implantation.	[20]
	Integrated disease cycle	Hyperinsulinemia and hyperandrogenism reinforce a pathological cycle of pro-inflammatory adipokine and chemokine secretion; disorders reflect convergent hormonal and immune dysregulation involving altered sex steroid receptor balance and impaired cytokine-mediated recruitment of natural killer cells.	[21,27-29]

Clinical symptoms and diagnostic characteristics

PCOS and endometriosis present distinct clinical manifestations and healthcare utilization patterns, as demonstrated by clinical and epidemiological studies, reflecting different symptom profiles and diagnostic pathways [31]. The most commonly reported symptoms in women with PCOS include fatigue, anxiety, and a body mass index greater than 25, whereas persistent lower abdominal pain with referred back pain is frequently reported in women with endometriosis. These symptom patterns, documented in clinical outcome studies, are commonly used in clinical assessments to guide diagnostic evaluation [31].

Psychosocial and developmental risk factors associated with these disorders have also been investigated in observational studies to better understand early-life influences on disease risk and diagnosis [32]. Associations between adverse childhood experiences and endometriosis have been reported; however, many of these findings rely on self-reported diagnoses rather than surgically confirmed histological evidence. This limitation reduces diagnostic certainty and highlights the importance of validated biomarkers and objective diagnostic tools in endometriosis research [32].

Healthcare Utilization and Diagnostic Delays

Differences in healthcare-seeking behavior further distinguish these conditions at the population level, as demonstrated by clinical service utilization data that influence the timing of diagnosis and disease recognition [33]. Outpatient specialist care data from Poland indicate that women with PCOS seek specialist consultation at an earlier mean age of 25 years, whereas women with endometriosis tend to present later, at an average age of 38 years. Such diagnostic delays may contribute to prolonged symptom burden and delayed clinical identification of endometriosis [33].

Developmental biomarkers also provide insight into the differing origins of these disorders [34]. Anogenital distance, a lifelong indicator of prenatal androgen exposure, has been reported to be longer in individuals with PCOS and shorter in those with endometriosis. These observations suggest that anogenital distance may function as a developmental biomarker reflecting early endocrine influences on disease susceptibility [34].

Epidemiological Burden and Diagnostic Challenges

When considered within the broader burden of gynecological diseases, these findings highlight common diagnostic challenges in clinical practice [25]. Uterine fibroids represent the most common non-malignant gynecological condition worldwide and show the highest age-standardized incidence and prevalence among women of reproductive age [25]. Ovarian cancer illustrates the consequences of non-specific symptom presentation, as many cases are diagnosed at advanced stages and the five-year survival rate is approximately 35%, emphasizing the broader challenge of early detection across gynecological disorders [2].

The gut microbiome has also been proposed to function as an extension of the endocrine system, and microbial dysbiosis may disrupt metabolic and hormonal homeostasis, thereby influencing reproductive tract disorders. Emerging evidence suggests that microbial signatures may serve as potential biomarkers of reproductive health and disease susceptibility [9]. PCOS remains the most common endocrine and metabolic disorder among women of reproductive age, with an estimated global prevalence of 5-20% [11].

The contrasting clinical features, prevalence patterns, and diagnostic pathways of PCOS and endometriosis support their classification as potentially diametric reproductive disorders. Delays in diagnosis, together with age-dependent patterns of specialist care utilization, contribute substantially to the global burden of non-malignant gynecological diseases and underscore the need for improved biomarker-based diagnostic strategies and earlier detection approaches [18,30,34]. Table 3 summarizes the differences in prevalence, clinical symptoms, and developmental biomarkers between endometriosis and PCOS.

**Table 3:** Epidemiological, clinical, and developmental characteristics This table was created by the authors based on a synthesis of data from the cited references [1,4,6,18,25,29-34]. ACE: adverse childhood experiences; PCOS: polycystic ovary syndrome; BMI: body mass index; AGD: anogenital distance; AGDAC: anogenital distance (anterior clitoral surface to anus); AGDAF: anogenital distance (posterior fourchette to anus)

Disorder	Parameter	Description	References
Endometriosis	Prevalence & Demographics	Affects approximately 5-10% of reproductive-age women globally, with peak incidence between 25-45 years and later mean age at diagnosis; frequently associated with earlier menarche.	[25,33]
	Clinical Presentation	Persistent lower abdominal pain with referred back pain; chronic pelvic pain, severe dysmenorrhea, dyspareunia, dyschezia, infertility, shorter menstrual cycles, and accelerated follicular recruitment.	[29,31]
	Psychosocial/Developmental Risk (ACE)	Associations between adverse childhood experiences and endometriosis are frequently reported in self-reported diagnosis studies, but not observed when endometriosis is defined by surgically confirmed histological lesions.	[32]
	Healthcare Utilization	Women seek specialist consultation at an older mean age (~38 years) in outpatient specialist care data from Poland.	[33]
	Developmental Marker (AGD)	Significantly shorter anogenital distance (AGDAC and AGDAF), reflecting lower prenatal androgen exposure.	[18,30,34]
PCOS	Prevalence & Demographics	Affects 5-20% of reproductive-age women worldwide, with an earlier mean age at diagnosis; commonly associated with delayed menarche.	[1,4,6]
	Clinical Presentation	Fatigue, anxiety, and BMI exceeding 25.	[31]
	Healthcare Utilization	Women seek specialist consultation at a younger mean age (~25 years) in outpatient specialist care data from Poland.	[33]
	Developmental Marker (AGD)	Significantly longer anogenital distance (AGDAC and AGDAF), indicating higher prenatal androgen exposure.	[18,30,34]

Pregnancy, obstetric, and long-term cardiometabolic outcomes

Pregnancy-Related Complications and Obstetric Risk

Reproductive disorders such as PCOS and endometriosis can influence pregnancy outcomes and long-term cardiometabolic health and are increasingly investigated using clinical and metabolic indicators associated with pregnancy-related risk [35]. Pregnancies in women with a history of PCOS are associated with an increased risk of cardiometabolic complications, particularly gestational diabetes mellitus and preeclampsia, which reflect underlying metabolic vulnerability during pregnancy [35].

Infertility-related interventions are also recognized as obstetric risk factors that may affect pregnancy outcomes and placental function [36]. Although infertility diagnoses themselves increase baseline obstetric risk, exposure to assisted reproductive technologies may independently contribute to adverse pregnancy outcomes, including higher rates of placental abnormalities [36].

Long-Term Cardiometabolic Consequences

Beyond pregnancy, PCOS represents an important long-term metabolic risk condition [37]. Women with this disorder have a higher likelihood of developing insulin resistance, type 2 diabetes, and cardiovascular disease, highlighting the value of metabolic indicators such as insulin resistance in identifying long-term health risks [37].

Evolutionary life-history frameworks have suggested that PCOS and endometriosis may represent divergent reproductive phenotypes. PCOS has been associated with increased visceral adiposity and metabolic energy storage, whereas endometriosis has been linked to greater early reproductive investment, reflecting differences in metabolic and endocrine profiles [38].

Metabolic and Environmental Risk Modifiers

Additional context-specific factors also contribute to reproductive and metabolic risk [25]. High body mass index is a major global risk factor associated with increased mortality and disability-adjusted life years in several gynecological diseases and is widely used as a metabolic risk indicator in reproductive health research [25]. Mechanistically, obesity-related oxidative stress in PCOS may impair oocyte cytoplasmic maturation through spindle abnormalities and mitochondrial dysfunction, indicating cellular processes associated with reduced oocyte quality [1].

Preventive factors have also been investigated in reproductive health research [20]. Prenatal vitamin D status has been associated with a reduced risk of gestational diabetes, and vitamin D-related endocrine signaling has therefore been explored as a metabolic indicator related to pregnancy outcomes [20]. Disturbances in gut microbial homeostasis have also been linked to altered energy balance and increased risk of preterm birth, with microbial composition increasingly studied as a potential biomarker of metabolic and reproductive health [9].

Collectively, systemic insulin resistance, dysregulated adipokine signaling, iron-mediated ferroptosis in placental tissues, alterations in maternal testosterone levels during pregnancy, and risks associated with assisted reproductive technologies represent interconnected physiological processes contributing to reproductive and metabolic disease risk [5,30,35,36].

Epigenetic regulation, miRNAs, and cellular proliferation pathways

MicroRNA Signaling and Ovarian Dysfunction

Epigenetic regulation and non-coding RNA signaling represent important molecular mechanisms involved in ovarian dysfunction and abnormal cellular proliferation in PCOS and other reproductive disorders. These pathways are increasingly investigated as potential molecular biomarkers for disease detection and progression [39]. Exosomal miR-18b-5p derived from follicular fluid activates PI3K/Akt/mTOR signaling and may contribute to the pathophysiology of PCOS through inhibition of PTEN expression. This process influences insulin sensitivity and granulosa cell proliferation and suggests a potential role for miR-18b-5p as a circulating miRNA biomarker associated with metabolic dysfunction [39].

Long Non-Coding RNAs as Regulatory Biomarkers

Long non-coding RNAs also contribute to regulatory mechanisms involved in ovarian dysfunction. The long non-coding RNA CDKN2B-AS1 has been reported to be overexpressed in PCOS and may promote abnormal granulosa cell proliferation by acting as a molecular sponge for miR-181a. This regulatory interaction indicates its potential relevance as a molecular marker associated with ovarian dysfunction and altered cellular proliferation [40].

Epigenetic Biomarkers and Disease Detection

These molecular alterations are increasingly relevant for disease detection and monitoring because circulating nucleic acids and molecular signatures are being incorporated into biomarker-based diagnostic strategies [41]. Screening approaches in gynecological malignancies frequently utilize molecular subtype classification and Nottingham histological grading to evaluate cellular proliferation and invasive potential. These approaches illustrate the broader diagnostic value of early molecular dysregulation in reproductive tissues [41].

Delayed diagnosis of conditions presenting with non-specific symptoms, such as reproductive pain or dyspnea, has been associated with increased long-term morbidity and mortality. This diagnostic challenge highlights the importance of identifying reliable molecular biomarkers that may facilitate earlier detection and improved disease monitoring [42].

Shared Molecular Pathways in PCOS and Endometriosis

PCOS and endometriosis have been proposed as possible diametric disorders of the hypothalamic-pituitary-gonadal axis, characterized by contrasting developmental patterns of prenatal testosterone exposure and adult AMH levels that may function as endocrine biomarkers distinguishing these conditions [18]. Molecular convergence between these disorders is also suggested by shared genetic risk factors [23].

Both conditions have been associated with reduced expression of SYNE1 and DNM3 in endometrial tissue, contributing to structural and functional alterations and indicating potential genomic markers of disease susceptibility [23]. Persistent low-grade inflammation within ovarian tissue may exacerbate oxidative stress in the follicular microenvironment, promoting granulosa cell apoptosis and follicular atresia. These processes are reflected in inflammatory and oxidative molecular signatures associated with reproductive dysfunction [1].

Women with irregular menstruation have been reported to exhibit DNA hypomethylation patterns and miRNA expression profiles in ovarian tissue that resemble those observed in ovarian cancer. These findings suggest the presence of epigenetic signatures that may serve as indicators of disease progression and potential malignancy risk [2].

**Figure 2:**
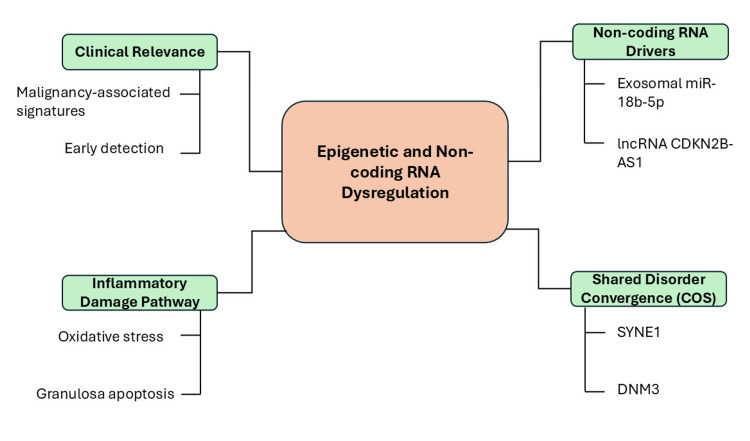
Epigenetic and non-coding RNA dysregulation in reproductive disorders Schematic representation of epigenetic and non-coding RNA dysregulation as a central mechanism linking molecular drivers, inflammatory damage pathways, and clinical relevance in disease progression. The diagram illustrates how non-coding RNA drivers, including exosomal microRNAs and long non-coding RNAs, contribute to regulatory disruption, while inflammatory damage pathways such as oxidative stress and granulosa cell apoptosis mediate tissue injury. These processes converge through shared molecular alterations, highlighting common disease-associated genes, and ultimately contribute to clinically relevant outcomes, including malignancy-associated signatures and early detection markers. miR: microRNA; lncRNA: long non-coding RNA; CDKN2B-AS1: cyclin dependent kinase inhibitor 2B antisense RNA 1; COS: convergence of shared disorders; SYNE1: spectrin repeat containing nuclear envelope protein 1; DNM3: dynamin 3 The figure was created by the authors of this study using Microsoft PowerPoint (Microsoft Corporation, Redmond, Washington, United States).

An integrated epigenetic network involving exosomal miRNAs and long non-coding RNAs may regulate metabolic homeostasis and granulosa cell proliferation, while shared genetic variants and pleiotropic loci contribute to the burden of non-malignant reproductive disorders through molecular pathways currently being explored for biomarker-based diagnostic applications [19,23,25,41]. Figure 2 illustrates the contribution of non-coding RNA dysregulation to ovarian dysfunction and disease progression.

AI in the early detection of cancer and complex diseases

AI Applications in Oncology as Methodological References

AI-based approaches have demonstrated considerable potential for early disease detection and risk stratification across multiple medical fields [43]. Several advances have been reported in oncology, where AI systems are increasingly applied to molecular and imaging data to improve diagnostic accuracy. For example, an AI system based on MethylBERT has demonstrated 80% sensitivity and 95% specificity for detecting early-stage epithelial ovarian cancer using circulating cell-free DNA (cfDNA) methylation markers obtained through liquid biopsy, supporting the feasibility of minimally invasive screening methods [43]. Similar methodological developments have also been reported in other malignancies [44]. In prostate cancer, AI analysis of multiparametric MRI combined with urinary exosomal biomarkers such as ExoDx Prostate IntelliScore has improved risk prediction accuracy [44].

Deep Learning for Imaging-Based Diagnostics

Deep learning techniques have also been widely applied in imaging-based diagnostics [45]. Convolutional neural networks enable automated detection and malignancy risk stratification of pulmonary nodules using CT and PET/CT imaging data [45]. In gynecological oncology and mammography, AI-based computer-aided detection systems improve the identification of microcalcifications, while AI-assisted Pap smear cytology enhances cell segmentation and classification accuracy. These developments illustrate how AI can assist clinicians by improving diagnostic precision and reducing manual interpretation errors [46].

AI-Supported Clinical Screening and Biomarker Discovery

Beyond imaging applications, AI integration can also improve the efficiency of diagnostic workflows and facilitate biomarker discovery [41]. Independent AI interpretation of mammography has been reported to increase cancer detection rates while reducing radiologist workload [41]. AI analysis of electrocardiogram data has also been shown to identify patterns associated with pulmonary hypertension several years before clinical diagnosis [42].

Bioinformatics platforms further support the discovery of miRNA biomarkers relevant to endocrine disorders such as PCOS [39]. In addition, machine-learning-based digital symptom checkers using least absolute shrinkage and selection operator (LASSO) regression have demonstrated approximately 78% accuracy in screening symptoms associated with PCOS, endometriosis, and uterine fibroids [31].

Overall, machine learning systems integrating radiomic, genomic, and clinical data have the potential to enhance diagnostic consistency, support earlier disease detection, and reduce clinical workload [44,46]. Although many of these applications have been developed in oncology and other medical specialties, they provide a methodological framework that may inform the development of AI-assisted screening approaches for reproductive disorders.

AI-driven screening, epidemiological modeling, and risk stratification in PCOS

Epidemiological Modeling and Disease Burden

AI-based screening and predictive modeling are increasingly being explored for risk stratification in PCOS [47]. AI-assisted optical microscopy enables automated and quantitative evaluation of reproductive parameters such as sperm concentration and motility, thereby improving objectivity in reproductive diagnostics [47]. Bayesian age-period-cohort modeling predicts that the global prevalence of PCOS may increase to approximately 3806 cases per 100,000 individuals over the next 40 years, indicating a growing epidemiological burden of the disorder [48].

Biomarker-Based Diagnostic Optimisation

Biomarker optimization may further improve diagnostic accuracy [49]. Age-specific screening thresholds for AMH have been reported to enhance diagnostic performance, with a critical value of 6.93 ng/mL identified for women aged 20-27 years [49]. These findings highlight the potential role of hormonal biomarkers in improving early detection and diagnostic precision in PCOS.

Clinical Risk Models and Digital Screening

Clinical risk modeling has also identified metabolic predictors associated with disease development [50]. Class III obesity and weight gain exceeding 20% have been reported as strong predictors of incident PCOS diagnosis [50]. Digital screening tools may further support early identification. Machine-learning-based symptom assessment using LASSO regression has demonstrated approximately 78% accuracy in detecting menstrual irregularities and hyperandrogenic symptoms associated with PCOS [31]. Mendelian randomization analyses also suggest a shared genetic architecture between PCOS and endometriosis, indicating overlapping biological mechanisms that may inform risk stratification models [23].

Advanced AI Architectures for Predictive Modeling

Advanced AI architectures are increasingly being investigated to enhance predictive modeling in reproductive health [43]. Transformer-based systems such as MethylBERT can analyze methylome-wide datasets to develop diagnostic models for gynecological conditions [43]. Risk stratification frameworks may also incorporate developmental perspectives, including the diametric disorder hypothesis, which proposes that PCOS and endometriosis represent contrasting developmental outcomes associated with prenatal testosterone exposure [18].

Effective risk stratification may require integration of epidemiological trends, metabolic risk indicators, molecular biomarkers, and AI-based analytical approaches to improve diagnostic accuracy and facilitate earlier detection of endocrine disorders such as PCOS [48-50].

Limitations and future directions

Although biomarker discovery and AI-based screening have advanced considerably, their direct application in reproductive disorders such as PCOS and endometriosis remains relatively limited. Many examples of AI-assisted diagnostics originate from oncology, including breast cancer, lung nodule detection, and prostate cancer screening. These examples are often cited to demonstrate methodological progress in machine learning rather than applications specific to reproductive medicine. While they illustrate the technical potential of AI-driven diagnostics, translation to reproductive health requires further validation using disease-specific datasets.

The current literature is characterized by heterogeneous study populations, cross-sectional designs, and reliance on surrogate or intermediate outcomes, which limit causal inference and reduce generalizability across populations with diverse genetic, environmental, and socioeconomic backgrounds. Inconsistent diagnostic criteria for endometriosis and PCOS further complicate dataset harmonization and model comparability, while reliance on self-reported symptoms may introduce measurement and recall bias. Several methodological limitations also affect the reliability of existing AI-based diagnostic models. Many algorithms lack external validation in independent populations, raising concerns about reproducibility and generalizability. Algorithmic bias may occur when training datasets are not demographically representative, potentially reducing diagnostic accuracy in underrepresented populations. In addition, the “black-box” nature of many machine learning models limits interpretability, which may reduce clinician confidence and hinder regulatory approval.

Ethical and regulatory challenges related to data governance, transparency, and clinical accountability also remain insufficiently addressed. These limitations suggest that, although AI-assisted diagnostics show promise, their integration into reproductive health screening frameworks requires careful validation, transparent model development, and clinically interpretable decision-support systems.

Future research should prioritize longitudinal, large-scale, and multi-ethnic cohort studies that integrate clinical, molecular, imaging, and digital phenotyping data to support the robust development and validation of predictive models. Establishing standardized biomarker datasets specific to reproductive disorders will be essential for translating AI methodologies that have proven effective in oncology into clinically relevant tools for reproductive health diagnostics.

## Conclusions

This narrative review highlights that endometriosis and PCOS are multifactorial reproductive disorders resulting from interactions among inflammatory, endocrine, metabolic, genetic, and epigenetic dysregulatory processes, with important implications for early diagnosis and risk assessment. The available evidence indicates limitations in symptom-based and single-modality diagnostic approaches and underscores the potential value of integrative models that combine molecular biomarkers, hormonal profiles, microbiome-related signals, imaging features, and digital phenotyping. Advances in AI show considerable potential to identify subtle preclinical patterns, improve diagnostic consistency, and reduce prolonged diagnostic delays that contribute to disease morbidity and progression. Multimodal and longitudinal models incorporating AI may enable scalable and personalized screening strategies aligned with biological and developmental risk profiles. However, translating these advances into routine clinical practice will require the development of explainable algorithms, standardized diagnostic definitions, and prospective validation in real-world clinical settings. Overall, the integration of systems biology with AI represents a promising approach for enabling earlier diagnosis, improving risk prediction, and supporting better reproductive health outcomes in individuals affected by endometriosis and PCOS.
